# Association study of nicotinic acetylcholine receptor genes identifies a novel lung cancer susceptibility locus near CHRNA1 in African-Americans

**DOI:** 10.18632/oncotarget.746

**Published:** 2012-11-16

**Authors:** Kyle M. Walsh, Christopher I. Amos, Angela S. Wenzlaff, Ivan P. Gorlov, Jennette D. Sison, Xifeng Wu, Margaret R. Spitz, Helen M. Hansen, Emily Y. Lu, Chongjuan Wei, Huifeng Zhang, Wei Chen, Stacy M. Lloyd, Marsha L. Frazier, Paige M. Bracci, Michael F. Seldin, Margaret R. Wrensch, Ann G. Schwartz, John K. Wiencke

**Affiliations:** ^1^ Department of Epidemiology and Biostatistics, University of California San Francisco, San Francisco, CA; ^2^ Department of Genetics, University of Texas M.D. Anderson Cancer Center, Houston, TX; ^3^ Department of Oncology, Karmanos Cancer Institute, Wayne State University, Detroit, MI; ^4^ Department of Genitourinary Medical Oncology, University of Texas M.D. Anderson Cancer Center, Houston, TX; ^5^ Department of Neurological Surgery, University of California San Francisco, San Francisco, CA; ^6^ Department of Epidemiology, University of Texas M.D. Anderson Cancer Center, Houston, TX; ^7^ Dan L. Duncan Cancer Center, Baylor College of Medicine, Houston, TX; ^8^ Department of Health Disparities Research, University of Texas M.D. Anderson Cancer Center, Houston, TX; ^9^ Department of Biochemistry and Molecular Medicine, University of California, Davis

**Keywords:** Lung cancer, nicotine dependence, African-Americans, genetic association, smoking

## Abstract

Studies in European and East Asian populations have identified lung cancer susceptibility loci in nicotinic acetylcholine receptor (nAChR) genes on chromosome 15q25.1 which also appear to influence smoking behaviors. We sought to determine if genetic variation in nAChR genes influences lung cancer susceptibly in African-Americans, and evaluated the association of these cancer susceptibility loci with smoking behavior. A total of 1308 African-Americans with lung cancer and 1241 African-American controls from three centers were genotyped for 378 single nucleotide polymorphisms (SNPs) spanning the sixteen human nAChR genes. Associations between SNPs and the risk of lung cancer were estimated using logistic regression, adjusted for relevant covariates. Seven SNPs in three nAChR genes were significantly associated with lung cancer at a strict Bonferroni-corrected level, including a novel association on chromosome 2 near the promoter of CHRNA1 (rs3755486: OR = 1.40, 95% CI = 1.18-1.67, P = 1.0 × 10^−4^). Association analysis of an additional 305 imputed SNPs on 2q31.1 supported this association. Publicly available expression data demonstrated that the rs3755486 risk allele correlates with increased CHRNA1 gene expression. Additional SNP associations were observed on 15q25.1 in genes previously associated with lung cancer, including a missense variant in CHRNA5 (rs16969968: OR = 1.60, 95% CI = 1.27-2.01, P = 5.9 × 10^−5^). Risk alleles on 15q25.1 also correlated with an increased number of cigarettes smoked per day among the controls. These findings identify a novel lung cancer risk locus on 2q31.1 which correlates with CHRNA1 expression and replicate previous associations on 15q25.1 in African-Americans.

## INTRODUCTION

Genome-wide association studies (GWAS) of lung cancer in European and East Asian populations have identified risk loci on chromosomes 5p15.33, 6p22.1-p21.31 and 15q25.1 [1-7]. Several single nucleotide polymorphisms (SNPs) on 5p15.33 and 15q25.1, which increase lung cancer risk in Europeans and Asians, have been shown to also increase risk for lung cancer in African-Americans [8-10]. Because African-Americans have higher lung cancer incidence rates and poorer lung cancer survival than other racial and ethnic groups in the United States [11], they are disproportionately affected by this disease. Population-level differences in the frequency of risk alleles may account for some of the differences observed in lung cancer incidence across ancestral groups [12]. Due to the presence of African admixture, African-Americans may also possess lung cancer risk alleles not found in Europeans [13]. Known racial differences in tobacco-related exposures, such as age when smoking is initiated, smoking intensity, attempts to quit and relapse behaviors, are also likely to contribute to observed racial differences in lung cancer incidence [14].

The lung cancer-associated region of 15q25.1 contains a cluster of three nicotinic acetylcholine receptor (nAChR) genes which have been associated with smoking behaviors, such as the number of cigarettes smoked per day [15] and nicotine dependence [16], suggesting that these variants influence cancer risk by augmenting smoking behaviors. There is also some suggestion that genetic variation in the region may influence lung cancer risk in a more direct manner, as some studies observed associations on 15q25.1 in never smokers [5, 17]. Furthermore, functional analyses have demonstrated that another gene on 15q25.1, *PMSA4*, may play a role in cancer cell proliferation and apoptosis [18]. Fine mapping of the associated region indicates that it is a lung cancer susceptibility locus in African-American populations [9], and that the risk observed in African-Americans may be greater than that in whites [10]. Further, there is some evidence that risk SNPs on 15q25.1 may have a more direct effect on lung cancer risk in African-Americans than in Europeans, where the SNPs do not contribute to cancer risk solely by increasing nicotine dependence [12].

While only the nAChR genes on 15q25.1 (*CHRNA5, CHRNA3, CHRNB4*) have been previously associated with lung cancer risk, there is strong biological rationale to believe that other nAChR genes may influence risk. The nicotinic acetylcholine receptors mediate the effects of nicotine in the body [19, 20]. These receptors are assembled from sixteen known genetically encoded nAChR subunits. The subunits, and the receptors assembled from them, fall into two classes: neuronal and neuromuscular. In humans the neuronal subunits consist of α2 through α7, α9, α10, and β2 through β4; the neuromuscular subunits are α1, β1, δ, ε and γ [21]. Variation in any one of these subunits could affect receptor function and influence smoking behavior and cancer risk.

We therefore conducted a comprehensive association study of 378 SNPs in the sixteen human nAChR genes using African-American lung cancer cases and controls recruited from three study centers. Here we report the results of these association analyses, focusing specifically on SNP associations in the thirteen nAChR genes not previously associated with lung cancer susceptibility.

## MATERIALS and METHODS

### Ethics Statement

All participating institutions received IRB approval, and appropriate written informed consent was obtained from human subjects.

### Study Population

A three center case-control study included African-American lung cancer cases and controls from three collaborating institutions: The MD Anderson Cancer Center (MDA) (479 cases, 376 controls), Wayne State University (WSU) (459 cases, 460 controls), and The University of California, San Francisco (UCSF) (447 cases, 453 controls). All study participants reported being of African-American ethnicity.

MDA cases were recruited from The University of Texas M. D. Anderson Cancer Center and the Michael E. DeBakey VA Medical Center, both in Houston. All cases with newly diagnosed, histologically confirmed lung cancer were eligible for the study. Case exclusion criteria were: prior chemotherapy, radiotherapy, or recent blood transfusion. African-American controls were recruited from Houston area community centers and the Kelsey-Seybold Foundation, Houston's largest multi-specialty physician group practice. MDA controls were matched to the cases on age (±5 years), sex, and African-American ethnicity.

Wayne State University cases were identified through the population-based Metropolitan Detroit Cancer Surveillance System, an NCI-funded SEER registry, as part of the EXHALE study [13]. Rapid case ascertainment was used to identify histologically-confirmed cases within several months of diagnosis. African-Americans diagnosed with a first primary lung cancer from November 1, 2005 through June 30, 2010 were recruited for the study. Controls were gathered through community-based recruitment and were frequency matched on age (±5 years), sex, and African-American ethnicity.

UCSF cases and controls were enrolled as part of The Northern California Lung Cancer Study, which has been described in detail elsewhere [22]. Cases and controls older than 18 years of age were identified during two collection periods, spanning September 1998–March 2003 and July 2005–March 2008. Cases were Northern California residents presenting with previously untreated, histologically confirmed lung cancer. Cases in the first accrual period were identified primarily through the Northern California Cancer Center (NCCC) rapid case ascertainment program and Alta Bates/Summit Hospital. Cases in the second accrual period were identified through both the NCCC and the Kaiser Permanente Medical Care Program (KPMCP). Control participants ascertained in the first accrual period were recruited through three sources: random-digit dialing, Health Care Financing Administration records, and community-based recruitment. Controls in the second accrual period were recruited through the KPMCP. UCSF controls were frequency matched on age (5 years), sex, and African-American ethnicity.

### Collection of covariates

Cases and controls recruited at all study sites completed interviews conducted by trained interviewers. Data on sex, age and smoking behaviors (i.e. “pack-years” and “cigarettes per day”) were collected as covariates for this study. *Never smokers* were defined as those who had smoked <100 cigarettes in their lifetime; *former smokers* were those who had quit smoking >1 year before diagnosis (cases) or interview (controls); *current smokers* included those who had quit smoking within the past 12 months. Pack-years for former and current smokers were calculated as the years smoked times the average number of cigarettes per day, divided by 20.

Cancer histology was determined using ICD-O codes abstracted from SEER (Surveillance Epidemiology and End Results) data from the California Cancer Registry (UCSF cases) or Detroit Cancer Registry (WSU cases). For MDA cases, tumor histology was abstracted from medical records. The following ICD-O groupings were made: adenocarcinoma (ICD-O: 8140, 8230, 8250-8255, 8260, 8310, 8333, 8470, 8480, 8481, 8490, 8550), squamous cell carcinoma (8052, 8070-8073, 8083, 8084), and small cell carcinoma (8041-8045).

### SNP Selection

The custom SNP panel included 120 ancestry-informative markers (AIMs) for the calculation of % African ancestry. The AIMs were chosen based on criteria to distinguish between different continental populations as previously described and empirically tested [23, 24]. An additional 378 SNPs included on the panel were selected to target the sixteen known nAChR genes. For a complete list of these SNPs, their NCBI37/hg19 positions, and the nAChR genes that they tag, see TableS1. SNP markers chosen for inclusion on the custom array were selected based upon the following criteria: known functional effect on activity of nicotinic acetylcholine receptors, validation in African or European populations, allele frequency > 0.05 in African populations, position across the region, predicted effect on function, r-square value with respect to other markers <70%, inclusion in one of three previous studies of African-American lung cancer susceptibility [8-10].

### Genotyping

MDA samples were genotyped at the MD Anderson Cancer Center using an Illumina Golden Gate Custom panel of 1536 SNPs. All MDA samples were unamplified genomic DNA derived from peripheral whole blood.

Wayne State University samples were genotyped at the Applied Genomics Technology Center (AGTC) at Wayne State University using the same Illumina Golden Gate Custom panel of 1536 SNPs. All WSU samples were unamplified genomic DNA, extracted from whole blood. Genotype reproducibility was verified with thirty duplicate samples, each with >99% concordance. Ten CEPH controls were genotyped and checked for concordance with published HapMap SNP genotypes at loci overlapping those assayed by the Illumina custom panel.

UCSF samples were genotyped at the University of California, San Francisco Genome Center using the same custom panel. Unamplified genomic DNA samples extracted from whole blood (n = 750) were genotyped along with whole genome-amplified (WGA) blood or buccal DNA samples (n = 150), prepared as previously described [25]. Genotypes for unamplified DNA and WGA DNA samples were clustered separately. Genotype reproducibility was verified using twelve duplicate samples with average concordance of 99.97% (99.68-100%). Ceph Trios were genotyped to assess the accuracy of assigned genotype clusters. The average heritability for nine Parent-Parent-Child trios was 99.88% (99.19-100%). All cluster plots were visually inspected.

For all three study sites, samples with genotyping call rate < 95% were excluded from analysis. SNPs with genotyping call rates <95% in any of the three sites were excluded from analysis of that site's data. To exclude poorly genotyped SNPs, any SNP with a Hardy-Weinberg Equilibrium (HWE) *X^2^* value >15 in controls, stratified by site, was removed from analysis of that site's data.

### Calculation of % African ancestry

The genetic structure of African-American study subjects was evaluated using the program Structure v2.3.1 [http://pritch.bsd.uchicago.edu/software] to estimate percent membership in three distinct founder populations: sub-Saharan African, European, and East Asian [26], where East Asian population ancestry was used as a surrogate for Amerindian descent. Founder population allele frequencies were defined using SNP data from 102 unlinked (r^2^<0.20) ancestry informative markers (AIMs), genotyped in 502 unrelated HapMap individuals (167 Yoruban Africans, 165 Caucasians, 84 Chinese and 86 Japanese) [27]. These same AIMs were genotyped in our study subjects for use with the Structure program.

### Statistical analysis of genotyped SNPs

Where the binary “case-status” variable was the outcome of interest, single SNP association statistics were calculated using logistic regression in Plink v1.07, assuming a log-additive model [http://pngu.mgh.harvard.edu/purcell/plink/] [28]. The effect of individual SNPs on lung cancer risk was calculated within each study site (MDA, WSU, UCSF) while adjusting for: sex, age and % sub-Saharan African ancestry. Modeling was also done with additional adjustment for number of pack-years smoked. Further modeling was conducted while stratifying by lung cancer histology (adenocarcinoma vs. controls, squamous cell carcinoma vs. controls, small-cell lung cancer vs. controls). Both these histology-specific analyses, and analyses of the full case-control dataset, were conducted within study sites and then combined across sites using fixed-effects meta-analysis using the standard error of the effect estimates to weight the studies. For all meta-analyses, the presence of heterogeneity across study sites was assessed using the Q-value, which was compared to a chi-square distribution on 2 degrees of freedom. Additionally, heterogeneity was qualitatively assessed using the I^2^ statistic [29]. All associations reported are for an allelic additive model, where odds ratios are for each additional copy of the minor allele. Associations are adjusted for: age, sex, % sub-Saharan African ancestry and, where indicated, for number of pack-years smoked.

Where the continuous “cigarettes per day” (CPD) variable was the outcome of interest, single SNP association statistics were calculated among ever smokers using linear regression in Plink v1.07. The effect of individual SNPs on the number of cigarettes smoked per day was calculated separately in lung cancer cases and in controls, while adjusting for: sex, age, study site and % African ancestry. Stratification by case-control status was done to prevent confounding of the SNP-CPD relationship by lung cancer status, and because the CPD variable was distributed differently in cases compared to controls. All associations reported are for an allelic additive model, adjusted for the indicated covariates.

### Correction for multiple testing

A total of 1536 SNPs were genotyped on the custom panel, of which 378 tagged nAChR genes. The remaining SNPs included 120 ancestry-informative markers, 494 SNPs used for fine-mapping of known GWAS hits at 5p15.33 and 6p22.1-p21.31, and 544 SNPs to be used for admixture mapping projects. A Bonferroni correction for the 378 nAChR SNPs on the array is overly conservative due to LD among SNPs. However, estimating the number of truly independent tests and using this as the correction factor results in only a minimal increase in the corrected value of α. As a result, we used Bonferroni correction, with a correction factor equal to the number of polymorphic SNPs successfully genotyped at 2 or more study sites. The Bonferroni-corrected significance threshold for this study is 1.49 × 10^−4^ (0.05/336).

### SNP imputation and association analysis in the CHRNA1 locus

To refine association peaks from the case-control analysis, we performed SNP imputation in regions that showed statistically significant association with lung cancer. However, no imputation was undertaken for the 15q25.1 region because, as a locus previously associated with lung cancer, the region was already blanketed with a large number of SNPs. As a result, imputation of SNPs in this region has limited value in our dataset because currently known SNPs were either directly genotyped or are well-tagged by directly genotyped SNPs (r-square > 0.80).

Imputation of a ~150kb region on Chromosome 2 containing the *CHRNA1* gene was performed with the Impute2 v2.1.2 software and its standard Markov chain Monte Carlo algorithm using the default settings for targeted imputation [http://mathgen.stats.ox.ac.uk/impute/ impute_v2.html#reference_2] [30]. All 1000 Genomes Phase I interim release haplotypes were provided as the reference haplotype panel [31]. Using a cosmopolitan set of reference haplotypes is currently recommended for imputation, and is especially critical for imputation of recently admixed populations like African-Americans [32].

Imputation was performed separately for each study site, and SNPs with imputation quality (info) scores < 0.70 or posterior probabilities < 0.90 were excluded to remove poorly imputed SNPs. Association statistics were calculated using logistic regression in SNPTEST v2 [http://mathgen.stats.ox.ac.uk/ genetics_software/snptest/snptest.html] assuming a log-additive model and using a missing data likelihood score test to account for genotype uncertainty. The effect of individual SNPs on lung cancer risk was calculated within each study site while adjusting for sex, age and % African ancestry. Site-specific effects were then combined across sites using fixed-effects meta-analysis as previously described. All associations reported are for an allelic additive model, adjusted for the indicated covariates, where odds ratios are for each additional copy of the minor allele.

### Ascertainment of imputation quality

To ensure accuracy of SNP imputation, a subset of 404 individuals (183 cases, 221 controls) from the UCSF arm of the study were genotyped at the most significantly associated imputed SNP using a TaqMan assay and the manufacturer's recommended protocol (Applied Biosystems; Foster City, CA). Thermal cycling was conducted and plates were read using a Bio-Rad CFX384 (Bio-Rad Laboratories, Hercules, CA) under recommended conditions.

To assess concordance of imputed genotypes with TaqMan genotypes, a weighted kappa statistic was calculated (κ_weighted_) to account for partial agreement occurring when heterozygotes were identified as homozygotes, and vice-versa. Such partial agreement was assigned a weight of 50% that of completely concordant genotypes. Odds ratios, p-values, and minor allele frequencies calculated using the imputed genotypes were also compared to those calculated using TaqMan genotypes.

### Relationship between SNP genotype and expression levels

Statistically significant SNPs associations identified in the case-control analysis of lung cancer patients were followed up to assess possible effects of the SNP on expression of nearby nAChR genes using publicly available expression data. The relationship between rs3755486 genotype and expression levels of *CHRNA1* in three cell types (primary fibroblasts, lymphoblastoid cell lines, and T-cells) was assessed in 85 individuals using the Illumina WG-6 v3 Expression array [33]. Online recovery and analysis of data was performed using the Genevar (GENe Expression VARiation) database and analysis tool [http://www.sanger.ac.uk/ resources/software/genevar/] [34]. Differences in the distribution of levels of mRNA expression between SNP genotypes were compared using Spearman's rank correlation coefficient (*rho*). A total of three expression probes (ILMN_2268969, ILMN_1798700, ILMN_2361768) targeted the *CHRNA1* gene, and significance thresholds were adjusted to account for a total of 9 comparisons (3 probes × 3 tissue types).

### Extension of novel SNP associations to individuals of European ancestry

Statistically significant SNPs associations identified in the case-control analysis of African-American lung cancer patients were followed up to assess possible effects in individuals of European ancestry using data culled from a previously published GWAS [6]. This dataset consists of 1154 cases with non-small cell lung cancer and 1137 controls, frequency matched on: age (±5 years), sex, smoking behavior, and years since quitting. Individuals were genotyped at 315,450 SNPs using Illumina HumanHap300 v1.1 BeadChips and standard GWAS methods, including adjustment for genomewide ancestry.

The associations of two SNPs near *CHRNA1* (rs3755486 and rs2244340) were evaluated in the European-ancestry case-control sample. Replication was not attempted for SNPs in the 15q25.1 region because the same region has previously been associated with lung cancer in this European-ancestry cohort [6].

## RESULTS

After excluding samples with call rates <95%, 2549 African-American subjects remained for analysis (1308 cases, 1241 controls). Compared to controls, cases were more likely to be male, to be older, to smoke, and to have smoked a greater number of pack-years and cigarettes per day (Table S2). Cases and controls had similar levels of sub-Saharan African ancestry and European ancestry (Table S2, Figure S1). After excluding monomorphic SNPs and SNPs that did not meet call-rate or HWE thresholds in at least two of the three study sites, 336 SNPs remained for analysis.

Seven SNPs were significantly associated with lung cancer risk at a Bonferroni-corrected level (P < 1.49 × 10^−4^) (Table 1). One of these is a novel association detected near *CHRNA1* on Chromosome 2q31.1. The other six SNPs are located in the 15q25.1 region previously associated with lung cancer. None of the SNP associations showed statistically significant heterogeneity across study site, as measured by Cochran's Q or I^2^.

**Table 1:** Significant SNP associations from the case-control analysis of lung cancer in African-Americans

				MD Anderson^c^		Wayne State^c^		UCSF^c^		Combined^d^		
SNP^a^	CHR	Gene	Allele^b^	OR	P-value	OR	P-value	OR	P-value	OR	P-value	MAF^e^
rs17486278	15	intron 1 of CHRNA5	A/C	1.32	0.014	1.46	8.5×10-3	1.34	4.4×10-3	1.37	6.4×10-7	0.28
rs2036527	15	6.3kb upstream of CHRNA5	C/T	1.58	2.5×10-4	1.34	0.014	1.25	0.055	1.37	4.3×10-6	0.21
rs16969968	15	exon 5 of CHRNA5	G/A	2.07	9.1×10-4	1.46	0.066	1.43	0.057	1.60	5.9×10-5	0.06
rs7180002	15	intron 2 of CHRNA5	A/T	1.62	4.5×10-3	1.67	9.7×10-4	1.14	0.39	1.44	7.1×10-5	0.10
rs3755486	2	2.7kb upstream of CHRNA1	C/T	1.34	0.074	1.53	4.4×10-3	1.35	0.038	1.40	1.0×10-4	0.11
rs951266	15	intron 2 of CHRNA5	C/T	1.62	4.5×10-3	1.65	1.3×10-3	1.12	0.43	1.42	1.0×10-4	0.10
rs4243084	15	intron 1 of CHRNA3	C/G	1.47	4.8×10-3	1.39	6.9×10-3	1.16	0.22	1.33	1.1×10-4	0.18

a ordered by P-value in the combined analysis

b minor allele listed second

c OR for each additional copy of the minor allele, estimated in a logistic regression model adjusted for: age, sex, and % African Ancestry. P-value derived from this model.

d OR for each additional copy of the minor allele, estimated using fixed-effects meta-analysis and adjusted for: age, sex, and % African Ancestry. P-value derived from this model. I2 can range from 0-100, where larger numbers indicate a greater level of heterogeneity across sites.

e Minor Allele Frequency is among controls.

Located <3kb upstream of *CHRNA1*, rs3755486 showed a strong association with lung cancer risk (OR = 1.40, 95% CI = 1.18-1.67, P = 1.0 × 10^−4^). *CHRNA1*, a neuromuscular nAChR subunit gene, has not been previously associated with lung cancer risk or with smoking behaviors. Adjustment for number of pack-years smoked did not attenuate the rs3755486 association (OR = 1.42, 95% CI = 1.19-1.70, P = 8.7 × 10^−5^). Although rs3755486 did not show a statistically significant association with lung cancer risk among never-smokers (OR = 1.33, 95% CI = 0.84-2.12, P = 0.22), this subgroup includes only 126 cases and 388 controls and statistical power is severely diminished.

**Figure 1:**
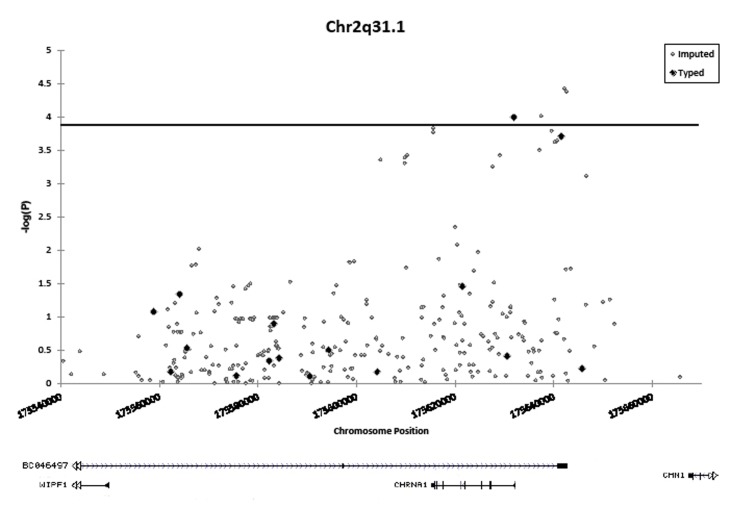
Association of 16 genotyped and 305 imputed SNPs on chromosome 2q31.1 with lung cancer in African-Americans The horizontal line indicates the study-wide Bonferroni-corrected significance threshold (1.49 × 10^−4^). Black diamonds denote SNPs which were directly genotyped on array. Gray circles denote imputed SNPs. Associations are adjusted for: sex, age, and % African ancestry, and combined across sites with fixed-effects meta-analysis.

Targeted imputation of an ~150 kb region on 2q31.1 containing the *CHRNA1* gene permitted calculation of association statistics for an additional 305 SNPs, three of which had smaller p-values than the most significant genotyped SNP (rs4972458, rs2646151 and rs935864) (Figure 1 and Table S3). While the most significantly associated genotyped SNP surpassed the Bonferroni threshold, the additional associations identified through imputation further indicate that the association peak is located near the *CHRNA1* gene promoter. The most significant of these imputed SNPs was rs4972458 (OR = 1.33, 95% CI = 1.16-1.52, P = 3.8 × 10^−5^).

Because SNP imputation in admixed populations, such as African-Americans, tends to be less accurate than in other populations, imputation accuracy was assessed with TaqMan genotyping. Imputed genotype at the rs4972458 risk SNP was strongly correlated with TaqMan genotype in a subset of 183 cases and 221 controls from UCSF undergoing genotyping at this locus (κ_weighted_ = 0.78, 95% C.I. = 0.72- 0.83; r = 0.83; 55/404 discordant genotypes). Odds ratios and p-values calculated from imputed genotypes were comparable to those calculated from TaqMan genotypes (OR = 1.53, P = 0.011 and OR = 1.45, P = 0.024, respectively). Further, the imputed minor allele frequency was similar to the minor allele frequency derived from TaqMan genotypes among cases (0.30 vs. 0.29, respectively) and among controls (0.21 vs. 0.22, respectively). Although we observed acceptable genotype concordance at the most significantly associated imputed SNP, it remains to be determined if the imputation was similarly accurate at neighboring markers.

**Figure 2:**
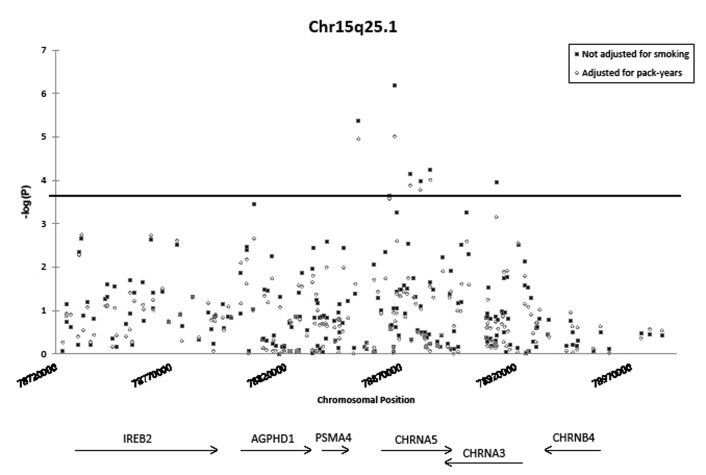
Association of SNPs on chromosome 15q25.1 with lung cancer, with and without adjustment for pack-years smoked The horizontal line indicates the study-wide Bonferroni-corrected significance threshold (1.49 × 10^−4^). Black squares depict association of SNPs with lung cancer, adjusted for: sex, age, and % African ancestry, and combined across sites with fixed-effects meta-analysis. Open circles depict association of SNPs with lung cancer, adjusted for: sex, age, % African ancestry, and number of pack-years smoked and combined across sites with fixed-effects meta-analysis.

Of the six significantly associated SNPs in the 15q25.1 region, the strongest association was observed at rs17486278 in intron 1 of *CHRNA5* (OR = 1.37, 95% CI = 1.21-1.55, P = 6.4 × 10^−7^). Of the significantly associated 15q25.1 SNPs listed in Table 2, only rs16969968 remained significantly associated with lung cancer when the model was conditioned on rs17486278 (OR = 1.36, P = 0.02). Likewise, only rs17486278 remained significantly associated when the model was conditioned on rs16969968 (OR = 1.22, P = 0.0050), indicating that multiple independent lung cancer risk loci may exist in the 15q25.1 region. Rs16969968 is a missense variant located in the fifth exon of CHRNA5 (OR = 1.60, 95% CI = 1.27-2.01, P = 5.9 × 10^−5^). This SNP is an A>G transition, changing an aspartic acid residue to an asparagine residue. No 15q25.1 SNPs in *CHRNB4* were significantly associated with lung cancer, but a single SNP in the first intron of *CHRNA3* did reach statistical significance (rs4243084, OR = 1.33, 95% CI = 1.15-1.53, P = 1.1 × 10^−4^). Adjustment for smoking modestly attenuated SNP associations on 15q25.1 (Figure 2). Furthermore, when analysis was restricted to never-smokers, rs2036527 remained associated with lung cancer risk (OR = 1.58, 95% CI = 1.12-2.26, P = 9.9 × 10^−3^).

**Table 2:** Associations of number of cigarettes smoked per day (CPD) with top ranked SNPs from the case-control analysis of lung cancer in African-Americans, stratified by lung cancer status

				Lung cancer cases^c^		Controls^c^	
SNP^a^	Position	Gene	Allele^b^	Effect±SE	P-value	Effect±SE	P-value
rs17486278	Chr15:78867482	intron 1 of CHRNA5	A/C	0.86±0.61	0.15	2.54±0.71	3.6×10-4
rs2036527	Chr15:78851615	6.3kb upstream of CHRNA5	C/T	0.089±0.67	0.89	2.69±0.81	9.7×10-4
rs16969968	Chr15:78882925	exon 5 of CHRNA5	G/A	0.44±1.05	0.68	3.15±1.49	0.035
rs7180002	Chr15:78873993	intron 2 of CHRNA5	A/T	−0.24±0.83	0.78	2.70±1.11	0.015
rs3755486	Chr2: 175631886	2.7kb upstream of CHRNA1	C/T	−1.49±0.81	0.065	−0.58±1.06	0.59
rs951266	Chr15:78878541	intron 2 of CHRNA5	C/T	−0.20±0.83	0.81	2.60±1.11	0.019
rs4243084	Chr15:78911672	intron 1 of CHRNA3	C/G	0.95±0.68	0.16	1.24±0.88	0.16

a ordered to match Table 2

b minor allele listed second

c Effect indicates the increase in CPD for each additional copy of the minor allele (lung cancer risk allele), estimated in a linear regression model adjusted for: age, sex, % African Ancestry, and study site. P-value derived from this model.

To explore whether the nAChR SNPs may influence cancer risk through modification of smoking behavior, associations between SNPs and the number of cigarettes smoked per day (CPD) were calculated separately among individuals with lung cancer and among controls. Five of the seven SNPs significantly associated with lung cancer in our study were also associated with CPD among the control subjects, with cancer risk alleles associated with heavier smoking. However, none of these SNPs were associated with CPD among lung cancer cases (Table 2). In controls, the strongest association with CPD was observed at rs17486278. This is also the SNP most strongly associated with lung cancer risk in the case-control analysis. For each additional copy of the lung cancer risk allele, an increase of 2.54 CPD was observed in the controls (95% CI = 1.15-3.94, P = 3.6 × 10^−4^).

To further explore the functional role of SNPs significantly associated with lung cancer risk, the relationship between genotype and gene expression level was assessed in three cell types. Functional data were not available for the 15q25.1 risk SNPs, but were available for rs3755486 on 2q31.1. *CHRNA1* mRNA expression, as measured by probe ILMN_1798700, was significantly associated with rs3755486 genotype in lymphoblastoid cell lines (ρ = −0.403, P = 3.0 × 10^−4^). The lung cancer risk allele was associated with increased levels of *CHRNA1* expression (Figure S2).

In an independent sample of 1154 European-ancestry cases with non-small cell lung cancer and 1137 European-ancestry controls collected at M.D. Anderson [6], rs3755486 was not associated with risk of lung cancer (OR = 1.01, 95% CI = 0.89-1.14, P = 0.896), nor was rs2244340. Associations were also null when the sample was restricted to cases with adenocarcinoma and to cases with squamous cell carcinoma.

## DISCUSSION

Our findings provide evidence that inherited variation in the nAChR genes influences lung cancer risk in African-Americans. We identified a novel risk locus on chromosome 2q31.1 which correlates with *CHRNA1* gene expression. Imputation of SNPs in the region identified additional associated variants and refines the association peak to the *CHRNA1* promoter region. We also replicate previously identified lung cancer associations in the nAChR gene cluster on 15q25.1. These associations are strongly localized to *CHRNA5* in our African-American sample, and the cancer-associated risk alleles in *CHRNA5* also influenced smoking behavior in our control population.

*CHRNA1* is a neuromuscular nAChR subunit gene. Although its expression was originally discovered in the neuromuscular junction, it has recently been detected in numerous other cell types, including: leukocytes, fibroblasts, epithelial cells and non-small cell lung cancer cells [35]. Analysis of both genotyped and imputed SNPs showed a significant association peak near the promoter of *CHRNA1*. The most strongly associated genotyped SNP in this region was highly correlated with *CHRNA1* gene expression, with increased copies of the risk allele associated with higher gene expression levels. The novel association observed at SNP rs3755486 near *CHRNA1* is especially intriguing because it accounts for a comparable proportion of lung cancer risk in African-Americans as the well-described missense variant rs16969968 on 15q25.1 (population attributable fractions of 4.21% and 3.48%, respectively).

Controlling for the number of pack-years smoked had only a minor effect on the *CHRNA1* associations. Because pack-years is an imperfect measurement of smoking behavior, it is certainly possible that the effects observed here are mediated by smoking. No significant associations were observed between *CHRNA1* SNPs and cigarettes per day, but this secondary outcome could only be evaluated in a subset of the sample and was not as well-powered as the case-control analyses. However, the two strongest *CHRNA1* associations in the full case-control dataset had consistent odds ratios when analysis was restricted to never-smokers (rs3755486: 1.40 vs. 1.33, rs2244340: 1.30 vs. 1.46).

The current analysis represents the largest genetic study of lung cancer conducted in African-Americans to date. While this is also currently the most thorough study of nAChR SNPs in African Americans, more comprehensive genotyping of nAChR genes in this population appears warranted. How risk alleles near the *CHRNA1* gene promoter may influence lung cancer incidence in African-Americans is particularly intriguing, as the allele identified as a novel risk factor tends to be more common in European populations (Figure S3). This allele is also detected in certain indigenous African populations, and therefore it may be found on multiple haplotype blocks of independent origin.

Studies conducted in additional ancestral populations will help to determine if *CHRNA1* SNPs confer lung cancer risk in multiple ethnicities, or if the effects identified here are specific to African-Americans. The most significantly associated *CHRNA1* SNP in the current study was not associated with increased lung cancer risk in a European-ancestry case-control sample. To better understand ethnic differences in the effects of *CHRNA1* SNPs, future studies may investigate if the risk alleles identified here rest on a different ancestral haplotype in African-American cases than in cases of European ancestry. Although complex questions remain about the influence of demographic and evolutionary history on lung cancer risk in African-Americans, our results extend current knowledge and identify a previously unreported and biologically relevant genetic association among this disproportionately affected population.

## FUNDING

This work was supported by National Institutes of Health grants: R01CA52689, R01ES06717, R01CA121197, R01CA121197S2, R01CA14176, R01CA060691, N01PC35145, HHSN261201000028C, and R25CA112355. The funders had no role in study design, data collection and analysis, decision to publish, or preparation of the manuscript.

## Supplementary Figures and Tables




