# Supplementary material for: Association study of nicotinic acetylcholine receptor genes identifies a novel lung cancer susceptibility locus near CHRNA1 in African-Americans
Source: Oncotarget. 2012 Nov 16;3(11):1428–38. doi: 10.18632/oncotarget.746 (PMC3717803; doi:10.18632/oncotarget.746)
Supplement: Supplementary file 1 [file oncotarget-03-1428-s001.pdf]

Association study of nicotinic acetylcholine receptor genes identifies a novel lung cancer susceptibility locus near CHRNA1 in African-Americans - Walsh et al

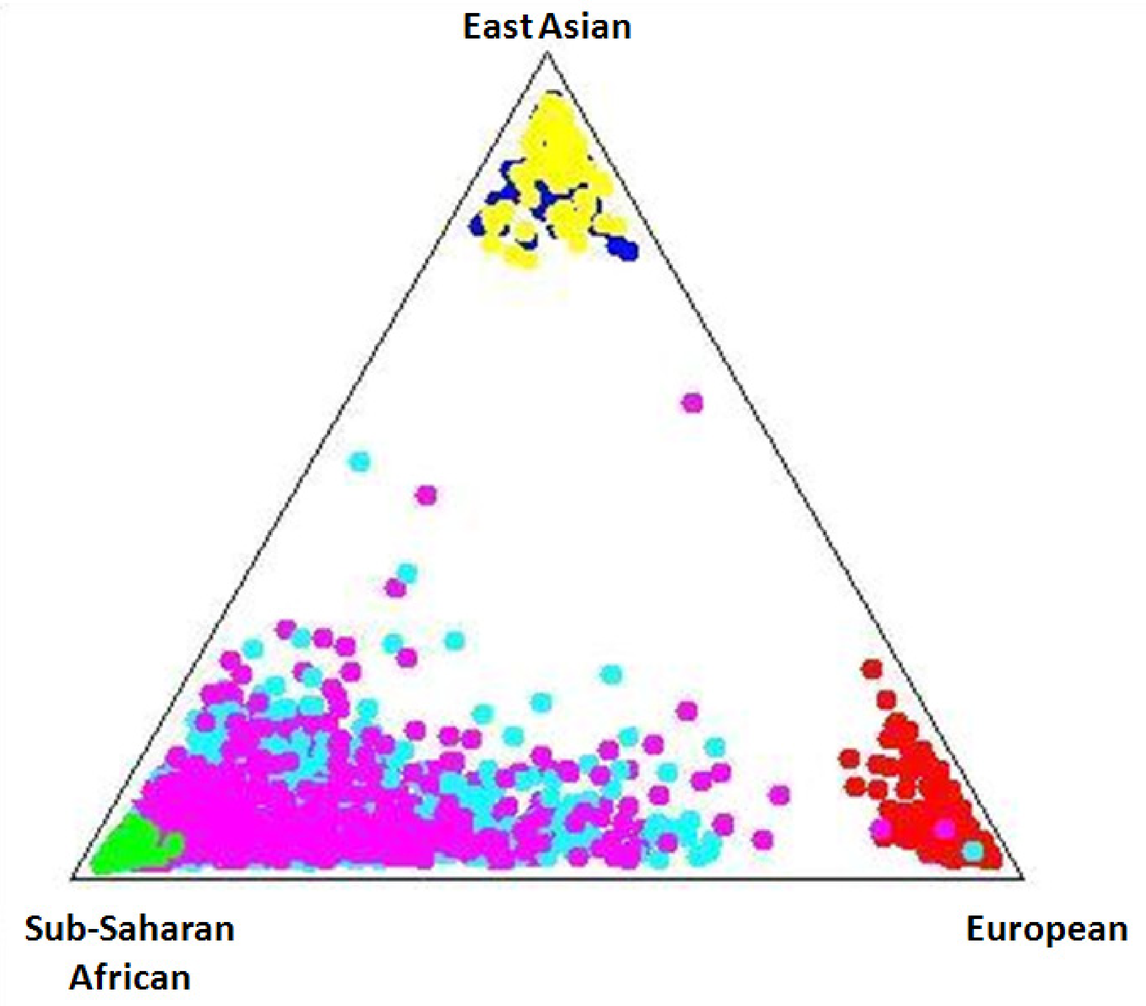

Figure S1: Triangle plot showing estimated admixture in the African-American case-control population. Estimates were performed using 102 AIMs and data from the International HapMap Project on 167 Yoruban African (Green dots), 165 Caucasian (Red dots), 84 Chinese (Yellow dots) and 86 Japanese (Dark Blue dots) founders. The figure depicts ancestry in 1308 African-American lung cancer cases (Pink dots) and 1241 controls (Light Blue dots).

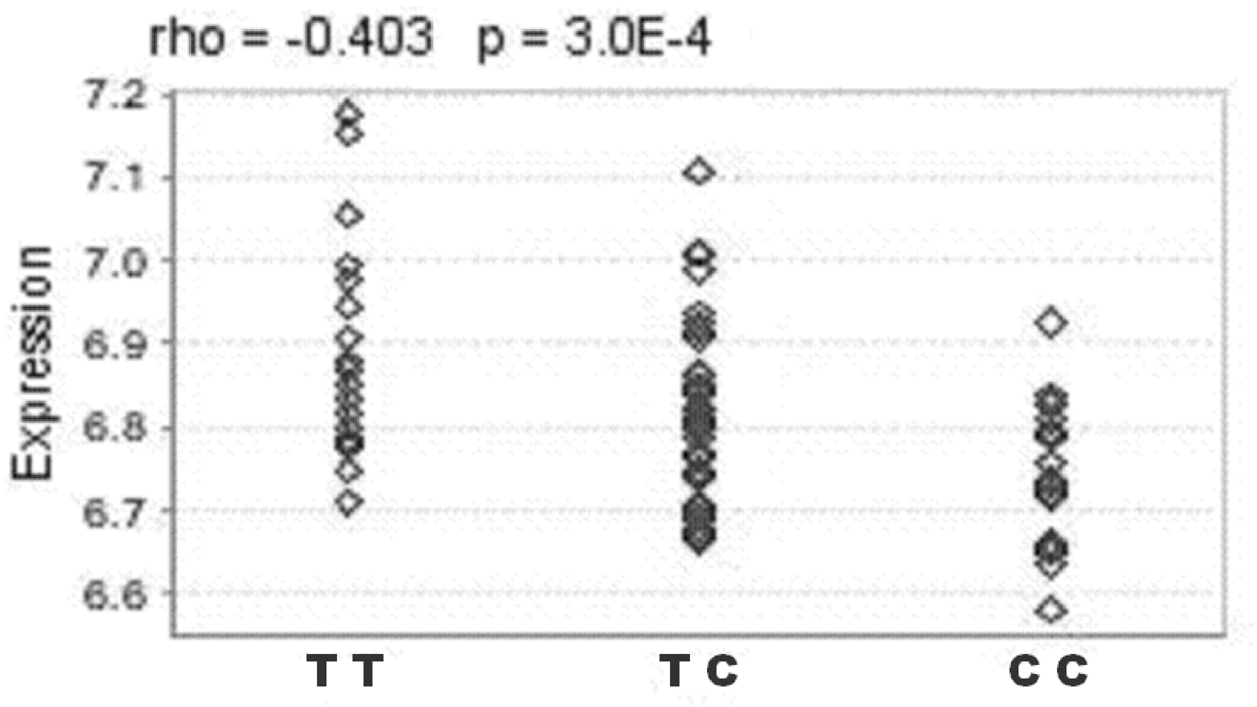

Figure S2: *CHRNA1* mRNA expression, as measured by microarray probe ILMN_1798700, in 85 lymphoblastoid cell lines stratified by genotype at SNP rs3755486 (Chr2:175631886). The minor allele (T) is associated with an increased risk of lung cancer and with increased *CHRNA1* gene expression ($\rho = -0.403$, $P = 3.0 \times 10^{-4}$). Data were recovered using the Genevar (GENe Expression VARiation) database and analysis tool.

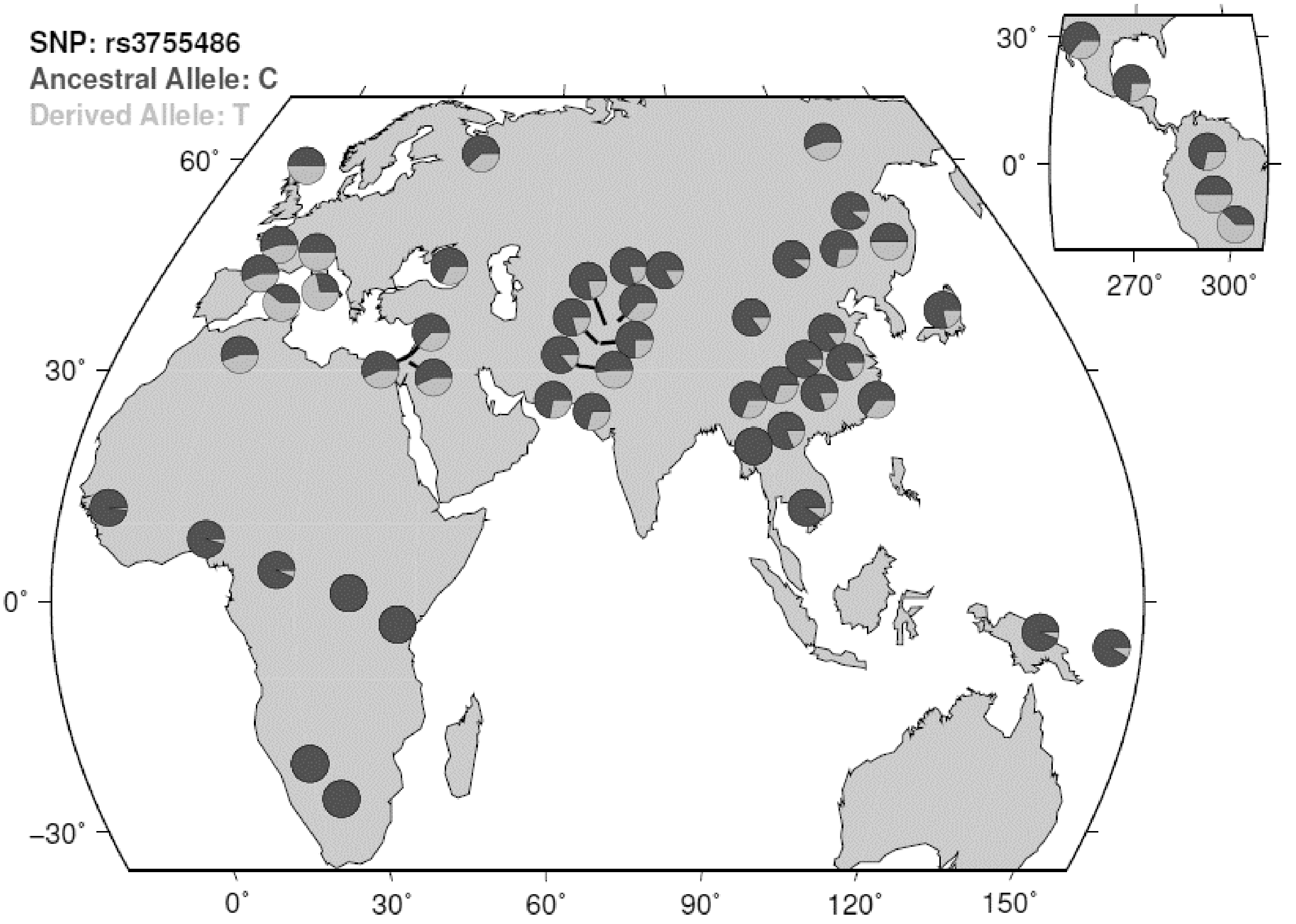

Figure S3: Global distribution of ancestral allele frequencies for a newly identified lung cancer risk SNP located near *CHRNA1* (rs3755486). The proportion of chromosomes possessing the ancestral allele (C) in a population is represented by the light gray sections of the pie charts. The proportion of chromosomes possessing the derived allele (T) in a population is represented by the dark gray sections of the pie charts. The derived allele is associated with an increased risk for lung cancer in our sample of African-Americans and has a minor allele frequency of 0.114 in our African-American controls. Data are abstracted from the Human Genome Diversity Project [http://hgdp.uchicago.edu/].
